# Lamin subtype expression in Merkel-cell carcinoma: its relation to polyomavirus infection and tumor metastasis

**DOI:** 10.1007/s00418-026-02526-1

**Published:** 2026-08-27

**Authors:** Merel Stiekema, Monique Ummelen, Moritz Jesinghaus, Corinna U. Keber, Viktoria Wischmann, Ingrid Moll, Jack Cleutjens, Marc A. M. J. van Zandvoort, Jos L. V. Broers, Frans C. S. Ramaekers, Roland Moll

**Affiliations:** 1 Department of Genetics and Cell Biology, Maastricht University Medical Centre, Universiteitssingel 50, 6229ER Maastricht, The Netherlands; 2 GROW—Research Institute for Oncology and Reproduction, Maastricht University Medical Centre, Maastricht, The Netherlands; 3 Institute of Pathology, Philipps-Universität Marburg, Marburg, Germany; 4 Department of Dermatology and Venerology, University Hospital Hamburg-Eppendorf, Hamburg, Germany; 5 Department of Pathology, Maastricht University Medical Centre, Maastricht, The Netherlands; 6 Cardiovascular Research Institute Maastricht (CARIM), Maastricht University Medical Centre, Maastricht, The Netherlands; 7 Institute for Molecular Cardiovascular Research (IMCAR), RWTH Aachen University, Aachen, Germany; 8 Institute for Mental Health and Neuroscience (MHeNS), Maastricht University Medical Centre, Maastricht, The Netherlands

**Keywords:** Nuclear lamina, Merkel cells, Immunohistochemistry

## Abstract

Merkel-cell carcinoma (MCC) is known for its high metastatic potential. Studies in several tumor types have proposed a role for up- or downregulation of nuclear A- and B-type lamins in facilitating the process of invasion and metastasis, but such studies are lacking for MCC. Therefore, lamin expression levels were examined in 38 tissue samples from 28 patients with MCC using immunohistochemistry. The series comprised primary tumors and different metastases and both Merkel cell polyomavirus (MCPyV) positive and MCPyV-negative cases. This study revealed that primary MCC tumors express variable, but mostly low levels of A-type lamins, while the B-type lamins are overall highly expressed. No obvious differences in lamin expression were observed between the MCPyV-positive and MCPyV-negative cases. A generally higher A-type lamin expression was detected in MCC metastases, although comparison of primary tumors versus metastases from the same patients showed either no change or up- or downregulation of A-type lamins. Normal Merkel cells were found to express both A- and B-type lamins. Conclusively, our data suggest a flexible A-type lamin expression in the process of MCC carcinogenesis and metastasis, which is an important finding regarding the aggressiveness of MCC and in the debate about the cellular origin of MCC.

**Supplementary Information:**

The online version contains supplementary material available at https://doi.org/10.1007/s00418-026-02526-1.

## Introduction

Merkel-cell carcinoma (MCC) is a rare but highly aggressive type of skin cancer with a high metastatic potential and poor prognosis (Arora et al. 2012). The carcinogenesis of MCC can be related to either the presence of clonally integrated Merkel cell polyomavirus (MCPyV) (MCPyV-positive tumor) or ultraviolet (UV)-mediated DNA damage (MCPyV-negative tumor) (Feng et al. 2008; Mohsen et al. 2024). Furthermore, immunosuppressed and older individuals are at higher risk of developing MCC. Although MCC expresses marker proteins that are also (specifically) found in Merkel cells of the skin (e.g., cytokeratin 20, NSE, chromogranin A, synaptophysin, CD56 etc.), the true origin of MCC remains unknown (Harms et al. 2018). Alternative hypotheses about the cellular origin of MCC have been proposed, one of which is their derivation from epidermal and dermal stem cells, dermal fibroblasts, and pre-/pro-B cells (Harms et al. 2018; Liu et al. 2016; Sauer et al. 2017). MCPyV-positive and -negative MCC share the same characteristic immunohistological markers, but these different subtypes of MCC can be distinguished on basis of MCPyV large T antigen (MCPyV-LT) immunostaining (Becker et al. 2017; Shuda et al. 2009). MCPyV-LT is one of the proteins that is important in the replication of the viral genome, while its truncated variant is required for MCPyV-mediated carcinogenesis (Harms et al. 2018; Krump and You 2021; Myrda et al. 2024).

MCC has a neuroendocrine phenotype and often shows an infiltrative growth pattern at its periphery, with extensive microscopically small invasions, not easily recognized by superficial inspection. This suggests that MCC cells exhibit a high migratory potential, which may be a result of changes affecting their cytoplasmic cytoskeletal integrity or nuclear structure, such as the nuclear lamina (Broers and Ramaekers 2014). The nuclear lamina, composed of the nuclear lamins and lamin-associated proteins, is a filamentous meshwork at the nucleoplasmic side of the nuclear envelope. Lamins are subdivided into A-type lamins (lamin A, lamin Adel10, and lamin C) and B-type lamins (lamins B1 and B2) and are involved in nuclear stability and many cellular processes, such as the breakdown and re-formation of the cell nucleus during cell division, DNA replication, and gene regulation (reviewed in de Leeuw et al. 2018; Sobo et al. 2024; Srivastava and Ehrlicher 2024; Stiekema et al. 2020). Additionally, lamins function as structural components by determining nuclear shape, and they support the stability and mechanical properties of the nucleus, but also of the cell as a whole (Broers et al. 2004). Furthermore, it has been demonstrated that the nuclear lamina is altered upon nuclear entry and/or exit by several types of viruses (reviewed in Stiekema et al. 2020). The nuclear lamins were also found to contribute to the replication of mouse polyomavirus and are important for its nuclear egress (Brustikova et al. 2024).

Differential lamin expression patterns have been reported in several different cancer types (Broers and Ramaekers 2014). Lamin expression patterns have been correlated with the aggressive nature of certain cancer types, such as the low expression levels of A-type lamins in small-cell lung cancer (SCLC), as compared with non-small-cell lung cancers (NSCLC), that express high(er) levels of A-type lamins (Broers et al. 1993). SCLC is an aggressive lung cancer with neuroendocrine properties and has a similar phenotype compared with MCC (Vilasi et al. 2024). Both SCLC and NSCLC display decreased lamin B1 levels (Broers et al. 1993; Jia et al. 2019), while upregulation of lamin B2 seems to stimulate cell migration in NSCLC (Zhang et al. 2020).

Lamins have been described to affect cancer progression through a variety of mechanisms, for example, by increasing cell proliferation, disturbing cellular signaling, and enhancing cell migration (Bell et al. 2022; Davidson and Lammerding 2014; Ho and Lammerding 2012; Zhang et al. 2020). The migration of cancer cells can be facilitated by either a low expression of A-type lamins, to make the nucleus more flexible (Broers et al. 2004; Fu et al. 2012), but also by a high expression of A-type lamins, to provide mechanical stability (Harada et al. 2014; Lammerding et al. 2006; Wintner et al. 2020). The in vitro study by Harada et al. (Harada et al. 2014), for example, demonstrated that cells with increased A-type lamin levels are not able to migrate due to a higher nuclear stiffness, whereas a reduction of A-type lamins was shown to decrease the rigidity of the nuclear envelope and therefore increased the flexibility of cells needed to penetrate the extracellular matrix. However, cells with very low levels of A-type lamins demonstrated higher apoptotic rates. Possibly, cancer cells make use of temporarily up- or downregulation of lamins to either withstand mechanical stress in the circulation (Mitchell et al. 2015), or invade other tissues (Bell et al. 2022).

Several of the aforementioned correlations suggest that stress-related changes in lamin expression patterns might also occur in the highly metastatic MCC, but no studies on the state of nuclear lamin expression of MCC have been published to date. In this study we therefore investigated the expression of the different lamin subtypes in a selection of primary MCCs and various types of MCC metastases. To this end, immunohistochemistry was performed for A- and B-type lamins on formalin-fixed, paraffin-embedded tissues.

## Materials and methods

### Tissue samples

#### MCC tissue samples

Formalin-fixed and paraffin-embedded (FFPE) human MCC tumor tissues were selected from the archives of the Institute of Pathology of the Philipps University of Marburg (Germany). The tumor tissues were obtained for routine histopathological diagnosis in the period between 2007 and 2019. The Ethics Committee of the Philipps University of Marburg provided permission for the use of these tissue samples for research purposes (reference number 43/21). The use of all patient material and patient data was also in agreement with the Dutch Code of Conduct for Health Research 2022 (Committee on Regulation of Health Research 2022). The tissue samples obtained from 30 patients include 18 primary tumors, 1 local recurrence, 14 lymph node metastases, 2 in-transit metastases, 1 liver metastasis, and 2 skin metastases. Paired cases are listed as “A” and “B” (Supplemental Table S1). Most samples were obtained by surgical resection, except for tissue material from some lymph node metastases that were obtained by needle biopsies. The diagnosis was performed independently by at least two experienced pathologists (R.M. and pathologists of the staff of the Institute of Pathology of the Philipps University of Marburg, Germany). Supplemental Table S1 summarizes the clinicopathological data of the MCC cases investigated in this study. The histopathological diagnosis was supported by immunohistochemical staining for typical MCC markers, i.e., cytokeratin 20, chromogranin A, and synaptophysin (Supplemental Table S1; for characteristics of the antibodies, see Supplemental Tables S2 and S3), using a Leica BOND-MAX automated immunostainer (Leica, Wetzlar, Germany) according to the manufacturer’s instructions.

#### Normal human skin tissue samples

Four formalin-fixed and paraffin-embedded (FFPE) normal head skin samples from the scalp of a 68-year-old woman, consisting of dermis and subcutaneous fat tissue with quite a lot of hair follicles were derived from the archives of the Institute of Pathology of the Philipps University of Marburg (Germany).

### Immunohistochemical (IHC) staining

#### Lamin and MCPyV-LT IHC

Sections of FFPE MCC 4 µm-thick tissue were heated on an 80 °C hot plate for 15 min, deparaffinized in xylol (3 × 10 min), and rehydrated in a descending alcohol series. For antigen retrieval, sections were boiled for 20 min in 10 mM Tris–HCl, 1 mM ethylenediaminetetraacetic (EDTA) buffer (pH 9.0) in a microwave oven and then left for 30 min at room temperature (RT) to cool down. After rinsing three times with MilliQ, sections were permeabilized by incubation in freshly prepared phosphate buffered saline (PBS) with 0.2% Triton X-100 (AppliChem, Darmstadt, Germany) for 10 min at RT, followed by washing for 5 min in PBS with 0.05% Tween 20 (PBST; Sigma, Burlington, USA). Subsequently, primary antibodies (Supplemental Table S2 for details and dilutions used) diluted in PBS with 1% bovine serum albumin (BSA; faction V fatty acid free; Roche, Basel, Switzerland) were applied to the FFPE sections and incubated overnight at 4 °C. After washing with PBST (2 × 5 min), the sections were incubated with secondary antibodies (Supplemental Table S3) diluted in PBS with 1% BSA for 30 min at 37 °C, followed by washing with PBST (2 × 5 min). The sections were incubated with avidin/biotin complex (ABC) (diluted 1:50 in PBS maximum 30 min before incubation; Vectastain Elite ABC-HRP Kit, Vector Laboratories, Burlingham, USA) for 30 min at 37 °C, followed by washing once with PBST and once with PBS. Next, the sections were incubated with freshly prepared diaminobenzidine (liquid DAB + substrate chromogen system, Dako, Glostrup, Denmark) for 7 min at RT and subsequently washed with MilliQ water to stop the reaction. Finally, the sections were counterstained with hematoxylin (hematoxylin solution modified according to Gill II, Merck, Darmstadt, Germany), dehydrated in an ascending ethanol series and mounted with Entallan (Merck, Darmstadt, Germany). The immunostained tissue sections were scanned using a slide digitalization system (Panoramic 1000; 3DHistech, Budapest, Hungary).

#### Lamin and cytokeratin 20 IHC double-label immunofluorescence

The 4 µm-thick FFPE normal skin tissue sections were pretreated as described in paragraph 2.2.1. Primary antibodies (Supplemental Table S4) were diluted in PBS with 1% BSA, applied to the FFPE sections and incubated for 1 h at 37 °C. After washing with PBST (2 × 5 min), the sections were incubated with secondary antibodies (Supplemental Table S5) diluted in PBS with 1% BSA for 30 min at 37 °C, followed by washing with PBST (2 × 5 min). The tissues were mounted with Tris-glycerol DABCO mounting medium (90% glycerol, 20 mM Tris–HCl pH 8.0, 2% 1,4-di-azo-bicyclo-2(2,2,2)-octane, and 0.02% sodiumazide) containing 0.5 µg/mL diamidino-2-phenylindole (DAPI; Sigma-Aldrich).

Confocal laser scanning microscopy (CLSM) imaging of the double-labeled skin sections was performed with a Leica SPE confocal with LAS AF software (Leica, Wetzlar, Germany) using an ACS APO 40×/1.15 oil lens or ACS APO 63×/1.30 oil lens, format of 1024 × 1024, and speed of 400 Hz. The secondary antibody used for the detection of CK20 was conjugated to FITC and was excited at 488 nm, and emission was detected at 498–560 nm. The Texas Red-conjugated antibodies used for the detection of lamins were excited at 532 nm and detected at 600–680 nm. For DAPI these settings were 405 nm and 435–540 nm, respectively.

Controls did not show cross-reactivity of the goat anti-mouse IgG2a secondary antibody, used for detection of CK20, with the primary antibodies used for the detection of lamins, nor did the goat anti-mouse IgG3 or IgG1 with the anti-CK20 primary antibody. Additionally, no bleed-through was seen between the FITC and Texas Red channels.

### Semi-quantitative analysis of lamin and MCPyV large T antigen IHC in MCC

**Fig. 1:**
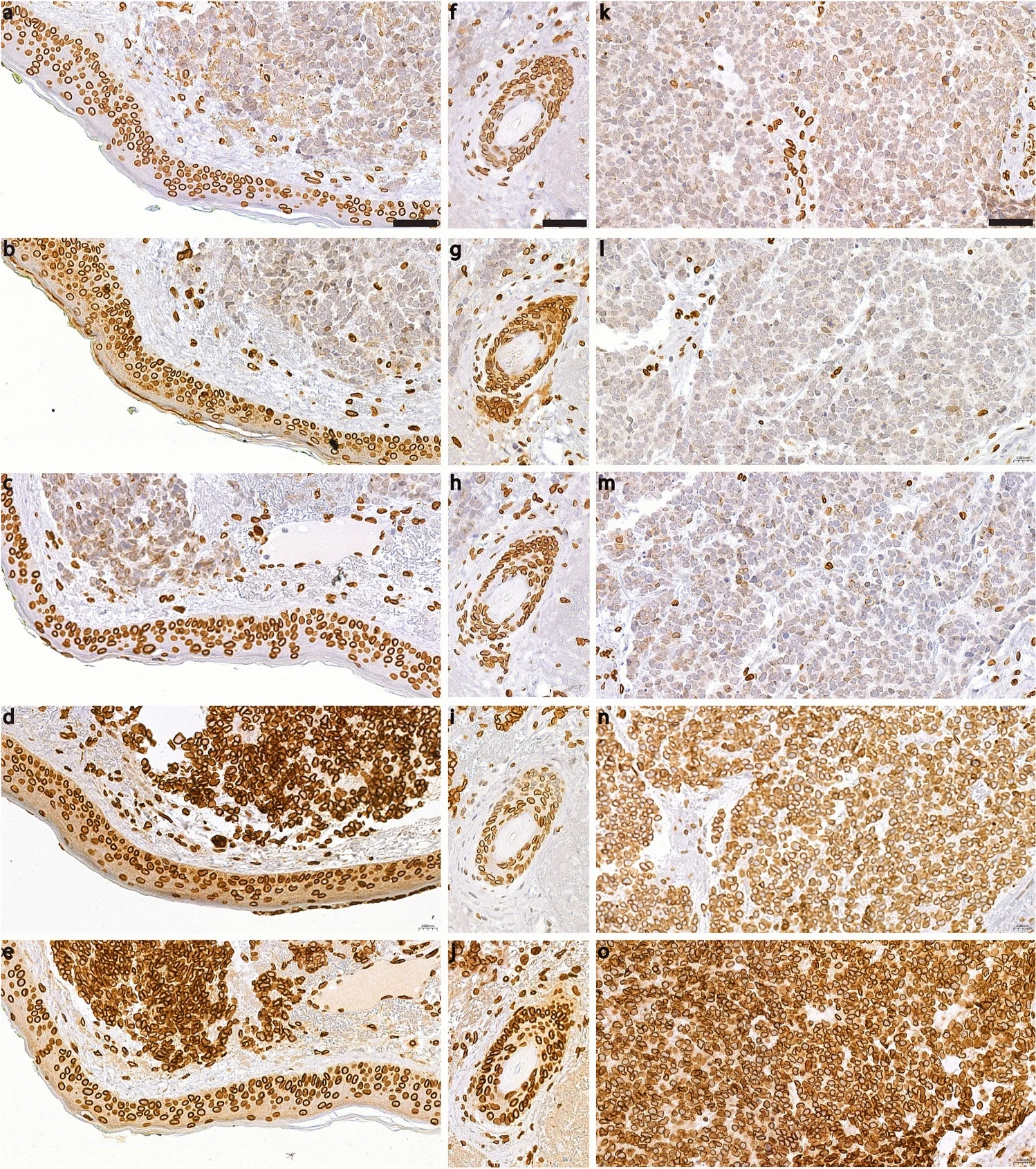
Lamin expression patterns in epidermis of case MC01 (**a**–**e**), and an epithelial skin appendage representing a small hair follicle (**f**–**j**), which were used as positive controls, next to the tumor tissue (**k**–**o**). Brightfield images of lamin A (**a**, **f**, and **k**), lamin C (**b**, **g**, and **l**), lamins A/C (**c**, **h**, and **m**), lamin B1 (**d**, **i**, and **n**), and lamin B2 (**e**, **j**, and **o**). Scale bars indicate 50 μm

Semi-quantitative analyses of the lamin expression levels were performed in the central part of the tumor tissue. The immunoreactivity score (IRS) was applied for the semi-quantitative analysis of all lamin subtypes (Remmele and Stegner 1987). The IRS was calculated by multiplying the percentage of positive cells (proportion score; 0–4 points) and the intensity score of the immunostaining reaction (0–3 points) (Table 1). Normal epidermis and epithelial skin appendages such as sweat glands, sebaceous glands, and hair root sheets, as well as stromal and endothelial cells were used as positive controls and the comparison of the intensity of immunostaining in these normal tissues to that in the MCC was used to determine the degree of positivity in the tumor tissue (Fig. 1).

**Table 1:** Overview of the indicators used for the immunoreactivity score (IRS) applied for the semi-quantitative analysis of A- and B-type lamin immunohistochemical staining. The IRS is determined by multiplying the score for the percentage of positive cells (0–4 points) by the staining intensity score (0–3 points)

Percentage of positive cells	Score	Staining intensity	Score
No positive cells	0	Negative staining (−)	0
< 10%	1	Weak staining (+)	1
10–50%	2	Moderate staining (+ +)	2
51–80%	3	Strong staining (+ + +)	3
> 80%	4

Semi-quantitative analysis of MCPyV-LT was performed by assessing the percentage of positive nuclei and classified into three different categories: “−” for cases with < 1% of the nuclei positive for MCPyV-LT, “+” for ≥ 1% and < 5% positive nuclei, and “+  +” for ≥ 5% positive nuclei.

## Results

**Fig. 2:**
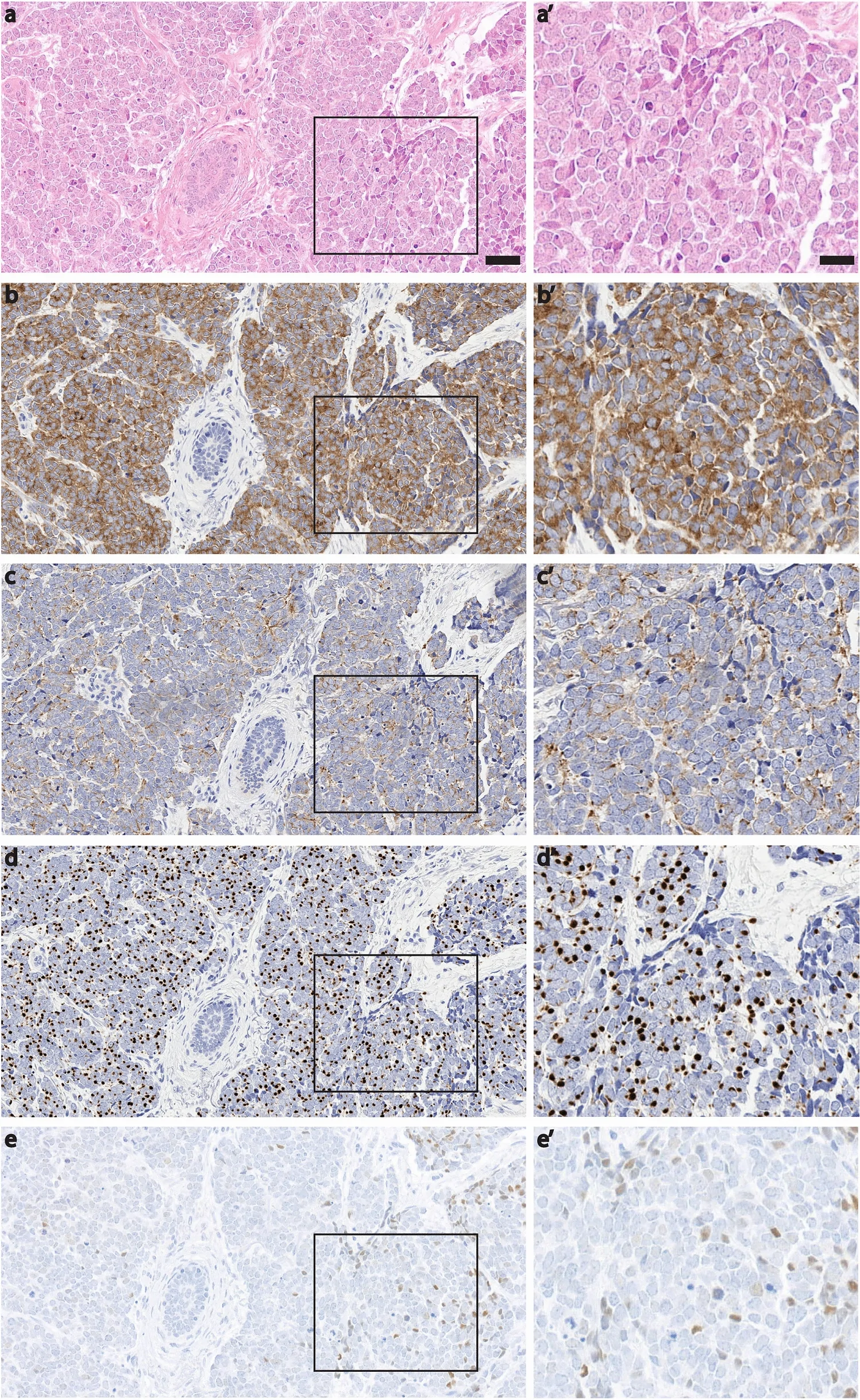
Neuroendocrine markers and MCPyV large T antigen (MCPyV-LT) in primary Merkel-cell carcinoma (case MC01). **a** Brightfield image of hematoxylin and eosin (H&E) staining. **b**–**d** Brightfield images of immunohistochemical markers of MCC: **b** synaptophysin, **c** chromogranin A, and **d** CK20. **e** Brightfield image of MCPyV-LT immunohistochemistry. **a′**–**e′** Higher magnification based on the region indicated in (**a**–**e**). Scale bar indicates 50 μm in overview images and 25 µm in higher magnification images

All MCC cases were tested for their expression of CK20 and the neuroendocrine markers chromogranin A and synaptophysin using IHC staining as part of routine diagnosis (Supplemental Table S1; examples of staining patterns for case MC01 in Fig. 2). The MCPyV-status for all cases was investigated using IHC of the MCPyV-LT-antigen, which revealed 23 of the 30 cases to be positive for MCPyV-LT-antigen (Supplemental Table S1), with variable positivity in both the number of positive cells and the intensity of staining (Supplemental Fig. S1).

The results described below are based on the analyses of the central parts of the MCC tissues. Occasionally, a stronger immunoreactivity was seen for A-type lamins at the margin of the tumor tissue or at the border to adjacent normal tissue or at the edge of the tissue section. One example of such an observation is presented in Supplemental Fig. S2. This effect was not observed for cytoplasmic constituents such as cytokeratin 20, the neuroendocrine markers, or the nuclear immunoreactivity of the MCPyV-LT antigen. However, for the nuclear mismatch repair protein MSH2, an enhanced staining intensity was observed at the tumor margin. Also, the nuclear proliferation marker Ki-67 did show a stronger immunostaining intensity at the margin of tumor nodules, which could not be correlated to an increased proliferative activity in these regions (results not shown).

### Low A-type and high B-type expression levels in primary MCC

**Fig. 3:**
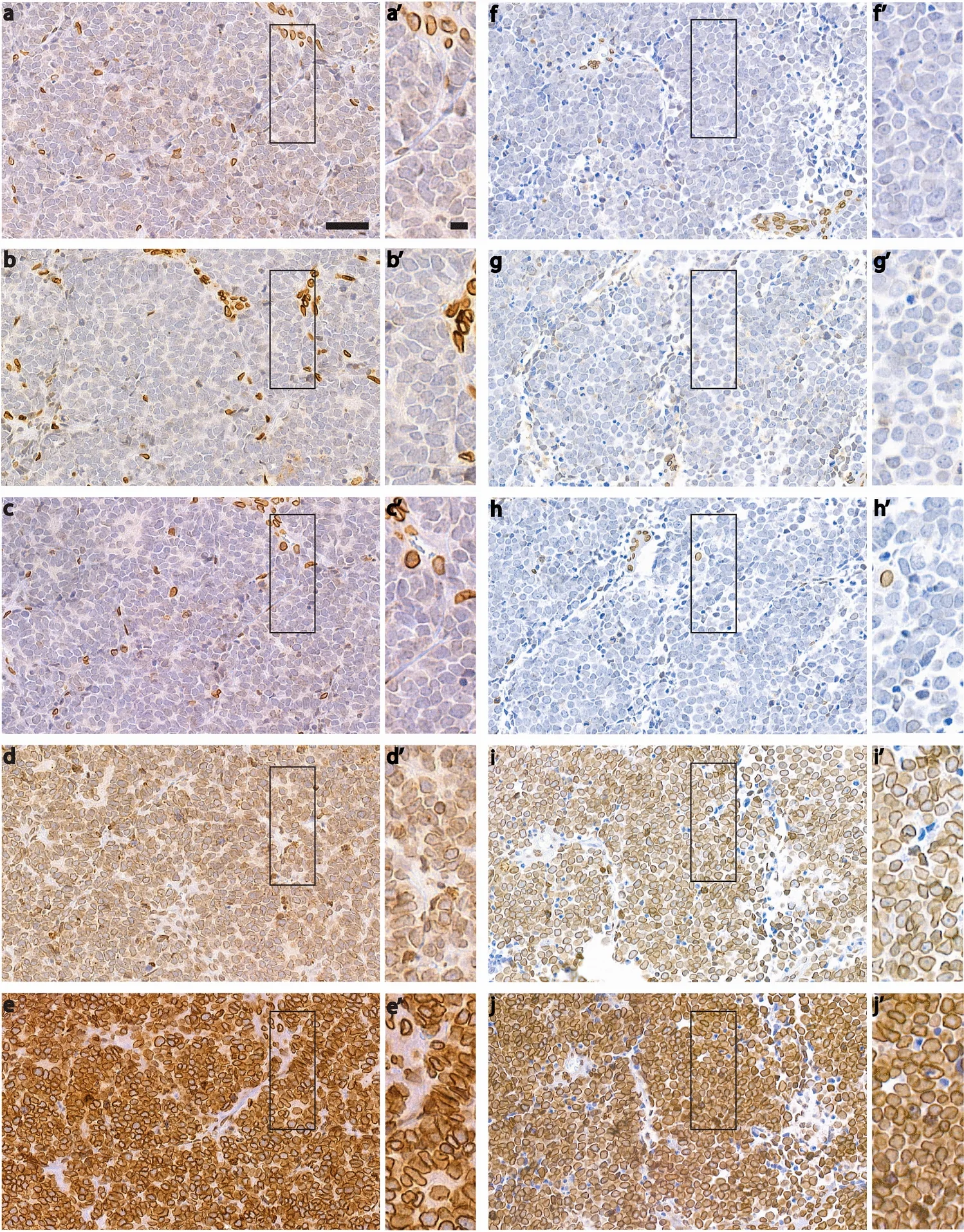
Low A-type and high B-type lamin expression in primary Merkel-cell carcinoma. **a**–**e** Case MC03. **f**–**j** Case MC13. Brightfield images of lamin A (**a** and **f**), lamin C (**b** and **g**), lamins A/C (**c** and **h**), lamin B1 (**d** and **i**), and lamin B2 (**e** and **j**) immunohistochemistry. **a′**–**j′** Higher magnification based on regions indicated in (**a**–**j**). Scale bar indicates 50 μm in overview images and 10 µm in higher magnification images

IHC staining of A-type lamins (using antibodies directed against only lamin A, only lamin C, or both lamins A and C) and B-type lamins (antibodies specifically directed against lamin B1 or lamin B2) revealed a differential nuclear envelope staining in primary MCC, with generally low expression of A-type lamins and a high expression of B-type lamins (Fig. 3), while control tissues were positive for all lamin subtypes (Fig. 1). The low expression level of A-type lamins was visible as both a low percentage of cells positive for the staining and a weak nuclear envelope staining in the positive cells (Supplemental Table S6), which in most cases results in an immunoreactive score (IRS) between 1 and 3 (Table 2). In the exceptional cases with a higher IRS, lamin A and lamin C were sometimes both high (e.g., MC11A), or only lamin A was expressed at a higher level (e.g., MC10 and MC28A). The IRS for both B-type lamins varied between 8 and 12, visible as a difference in staining intensity from moderate to strong staining (Table 2). Next to the primary tumors, a local recurrence was investigated, which displayed a similar A- and B-type lamin expression as most of the primary tumors (Table 2).

**Table 2:**
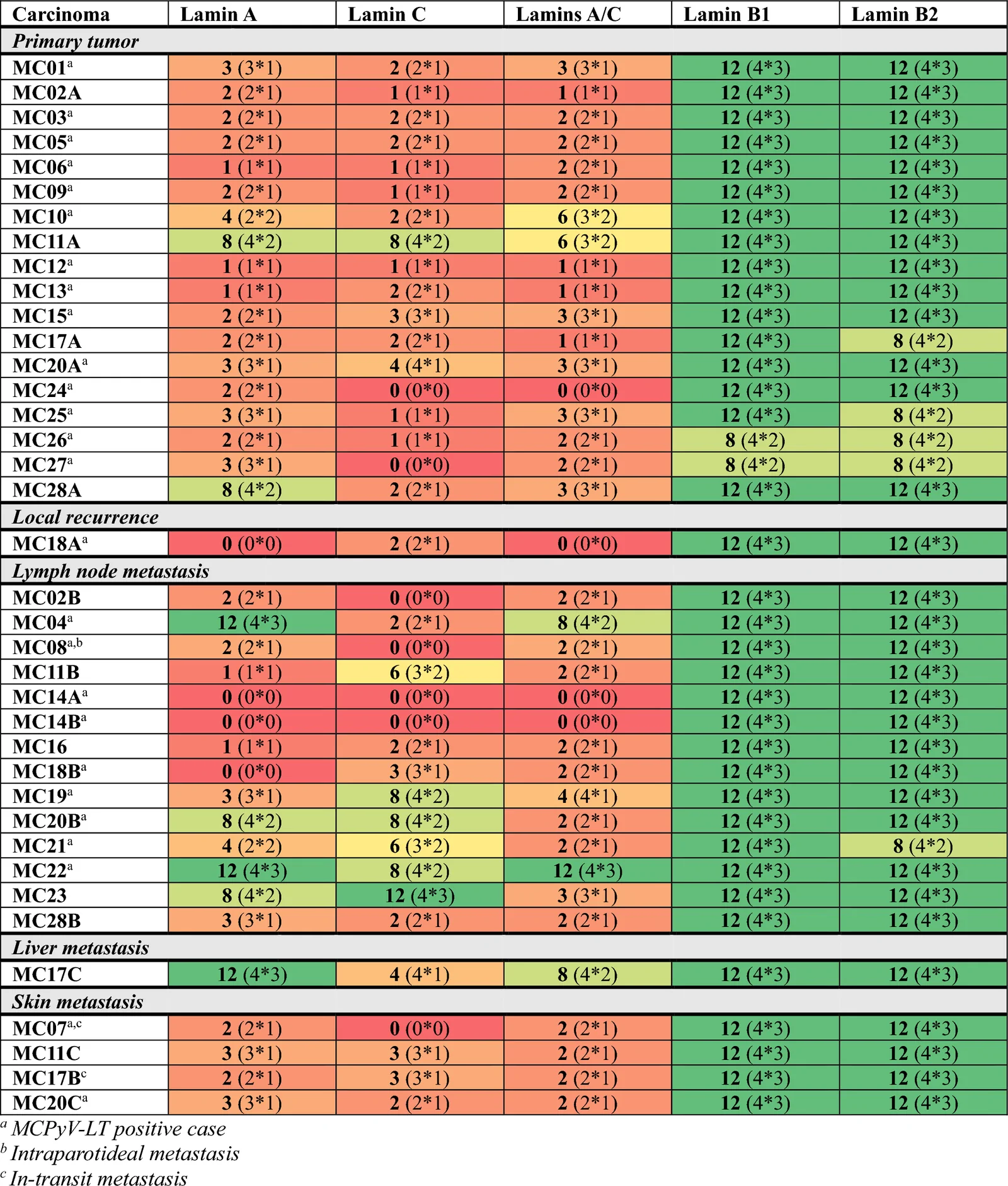
Overview of immunoreactive scores (IRS) for A- and B-type lamin immunohistochemical staining. The IRS is determined by multiplying the score for percentage of positive cells (0–4 points) with the staining intensity score (0–3 points); the score is represented as: total (percentage of positive cells × intensity score). The order of the cases has been rearranged to combine primary tumor samples and different types of metastases

^a^MCPyV-LT-positive case

^b^Intraparotideal metastasis

^c^In-transit metastasis

Comparing the differences in lamin expression to the MCPyV-status of the MCCs does not reveal clear differences between MCPyV-positive and MCPyV-negative samples, suggesting that the MCPyV-status does not have a direct impact on the lamin expression in primary MCC. Overall, these results reveal a generally low expression level for A-type lamins and high expression levels of B-type lamins in primary MCC.

### Flexible A-type lamin expression levels in MCC metastases

**Fig. 4:**
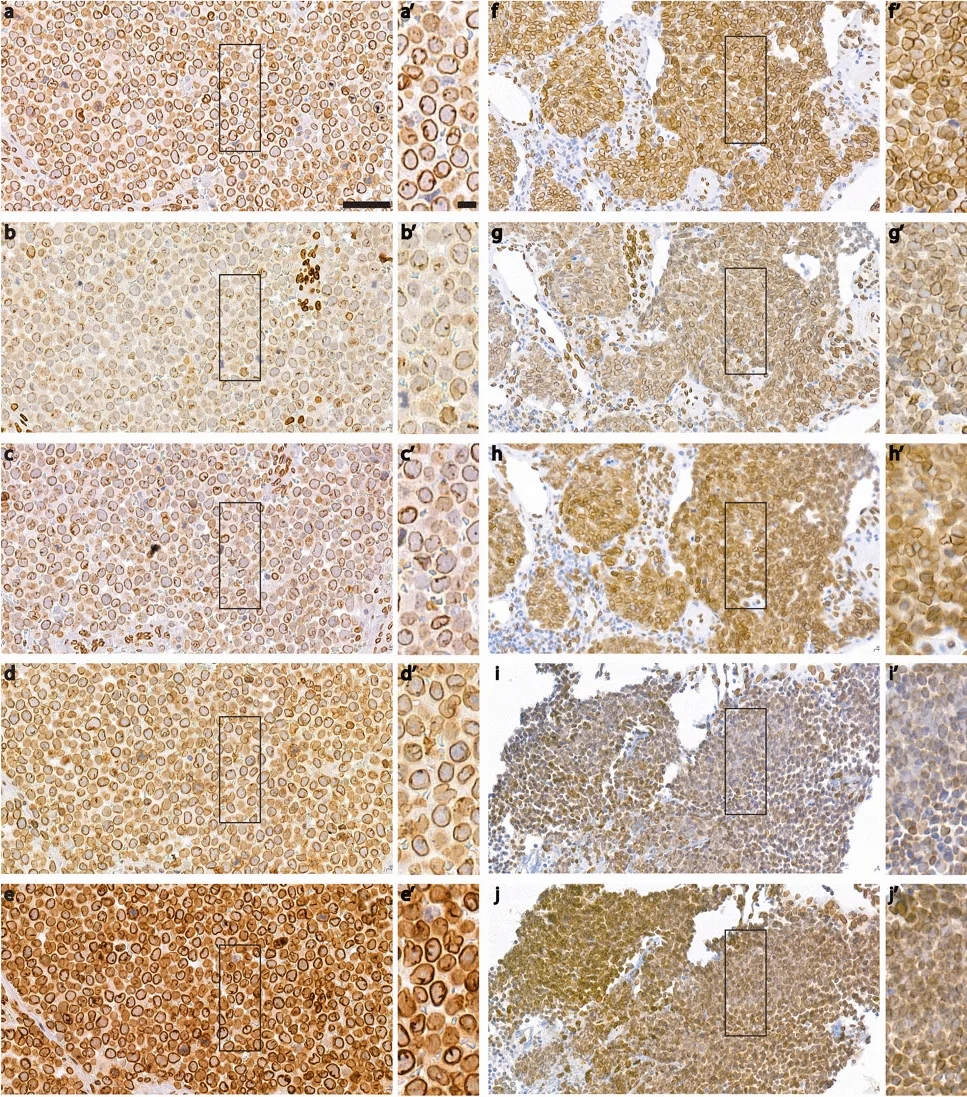
A- and B-type lamin expression in Merkel-cell carcinoma lymph node metastases. **a**–**e** Case MC04. **f**–**h** Case MC22. **i** and **j** Case MC20B. Brightfield images of lamin A (**a**, **f**, and **i**), lamin C (**b**, **g**, and **j**), lamins A/C (**c** and **h**), lamin B1 (**d**), and lamin B2 (**e**) immunohistochemistry. **a′**–**j′** Higher magnification based on regions indicated in (**a**–**j)**. Scale bar indicates 50 μm in overview images and 10 µm in higher magnification images

IHC staining of MCC tissues originating from lymph node metastases revealed in approximately half of the cases a higher expression level of A-type lamins as compared with the MCC tissues originating from primary tumors (Fig. 4a–c, f–j; Table 2). This difference was also reflected in the overall higher IRS and was visible as an increase in the percentage of cells positive for the A-type lamin staining, but in some cases also in combination with an increased staining intensity, ranging from moderate to strongly positive, such as cases MC22 and MC23 (Table 2; Supplemental Table S6). Despite the increased expression in the lymph node metastases, which seemed to be particularly true for lamin C, the A-type lamin expression levels were still clearly lower than B-type lamin expression levels. Both lamin B1 and lamin B2 revealed high expression levels in primary tumors (Fig. 3d–e, i–j) and metastases (Fig. 4d–e), which was reflected by an IRS between 8 and 12 (Table 2). However, while in a few cases of primary tumors a lower expression of B-type lamins, seen as an IRS of 8, was found, this only occurred once in the lymph node metastasis; case MC21 revealed an IRS of 8 for lamin B2.

The investigated MCCs included a few cases (with samples listed as “A” and “B” or “A”, “B”, and “C”) that originate from different locations of the same patient (Table 2; Supplemental Table S7). Between some primary and metastasis MCC tissues originating from the same patient, no to minor differences were seen in the A- and B-type lamin expression, such as for MC02. However, also frequently changes can be observed between the tissues of the same patient. For example, the lamin A expression in the primary tumor of MC28 was high (IRS of 8), but lower in the lymph node metastasis (IRS of 3). Strikingly, for case MC11, MC17 and MC20, the difference in lamin expression between primary tumor to metastasis was not similar for different types of metastasis. For example, in case MC11, a high A-type lamin expression was seen in the primary tumor, while this was low in the skin metastasis. For the lymph node metastasis, however, the lamin A expression was much lower (IRS of 8 in primary versus 1 in metastasis), but the lamin C expression was only slightly lower (IRS of 8 in primary versus 6 in metastasis) (Supplemental Fig. S3). In MC17, the primary tumor and skin metastasis revealed comparable A-type lamin expression, but in the liver metastasis this was much higher, with the IRS score of lamin A rising from 2 to 12 (Supplemental Fig. S4). Comparing the primary tumor and the skin and liver metastasis also demonstrated a small difference in the lamin B2 expression, with an IRS of 8 in the primary tumor and 12 in the metastases. These differences in paired cases seem to point toward a flexible lamin expression in MCC, especially for A-type lamins.

**Fig. 5:**
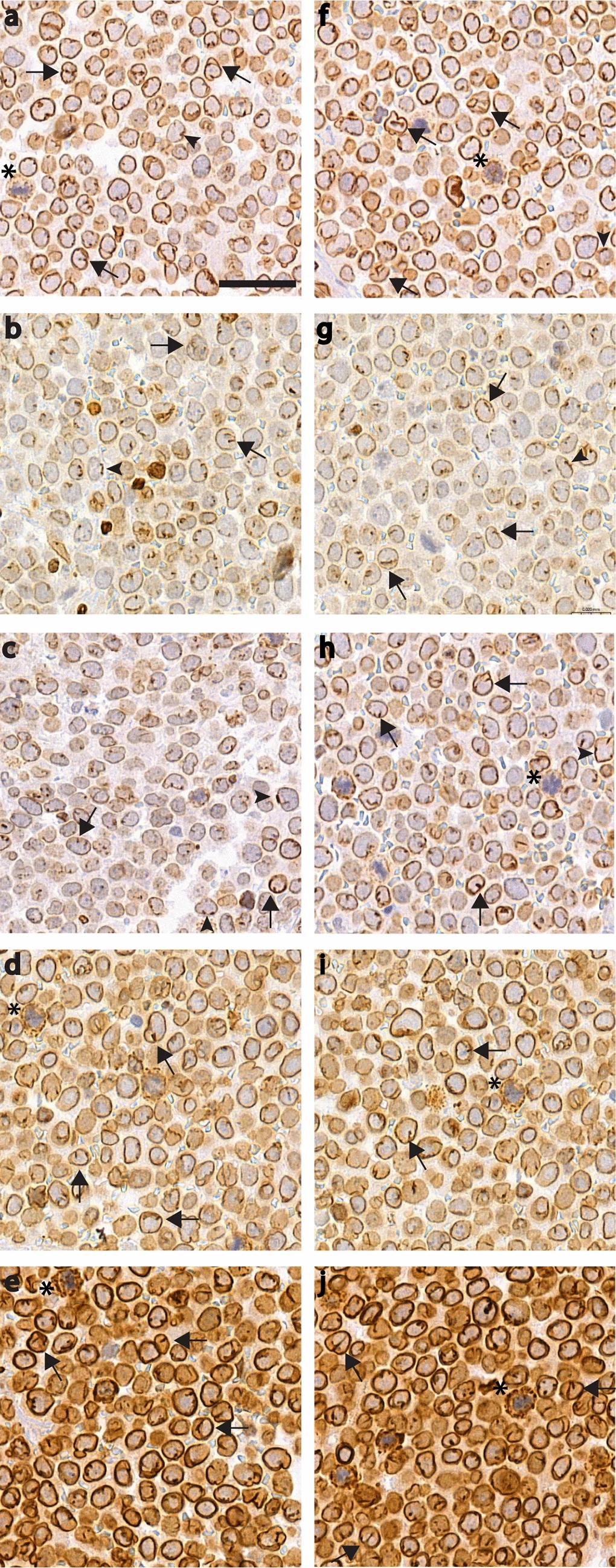
Cells in Merkel-cell carcinoma lymph node metastasis MC04 displaying particular, partly intranuclear immunostaining patterns of lamins, likely revealing lamin scars (arrowheads) and structures of the nucleoplasmic reticulum (arrows). In mitotic cells the lamins show the typical cytoplasmic lamin localization (asterisks) because of the disintegration of the lamina at this stage of the cell cycle. **a**–**j** Brightfield images of lamin A (**a** and **f**), lamin C (**b** and **g**), lamins A/C (**c** and **h**), lamin B1 (**d** and **i**), and lamin B2 (**e** and **j)** immunohistochemistry. Scale bar indicates 40 µm

In the stronger immunostainings of A-type lamins, other positive (sub)nuclear structures became apparent next to the staining of the nuclear lamina. Figure 5 illustrates these immunostaining patterns, which can be found for all the different lamin subtypes, but are more easily spotted in stronger A-type lamin staining. The enhanced immunoreactivity at regions of the nuclear lamina (arrowheads) resembles lamin scars formed after nuclear rupture, while the nucleoplasmic structures most likely represent tubules of the nucleoplasmic reticulum (arrows). In mitotic cells the disintegration of the nuclear lamina into the cytoplasm is visible (asterisks in Fig. 5).

Taken together these data demonstrate a general low A-type and high B-type lamin expression in primary MCC, while lymph node metastasis of MCC more frequently demonstrates higher A-type lamin expression. However, comparing paired cases reveals flexibility in the A-type lamin expression that can be either higher or lower in metastasis compared with the primary tumor.

### Lamin expression in normal human Merkel cells

**Fig. 6:**
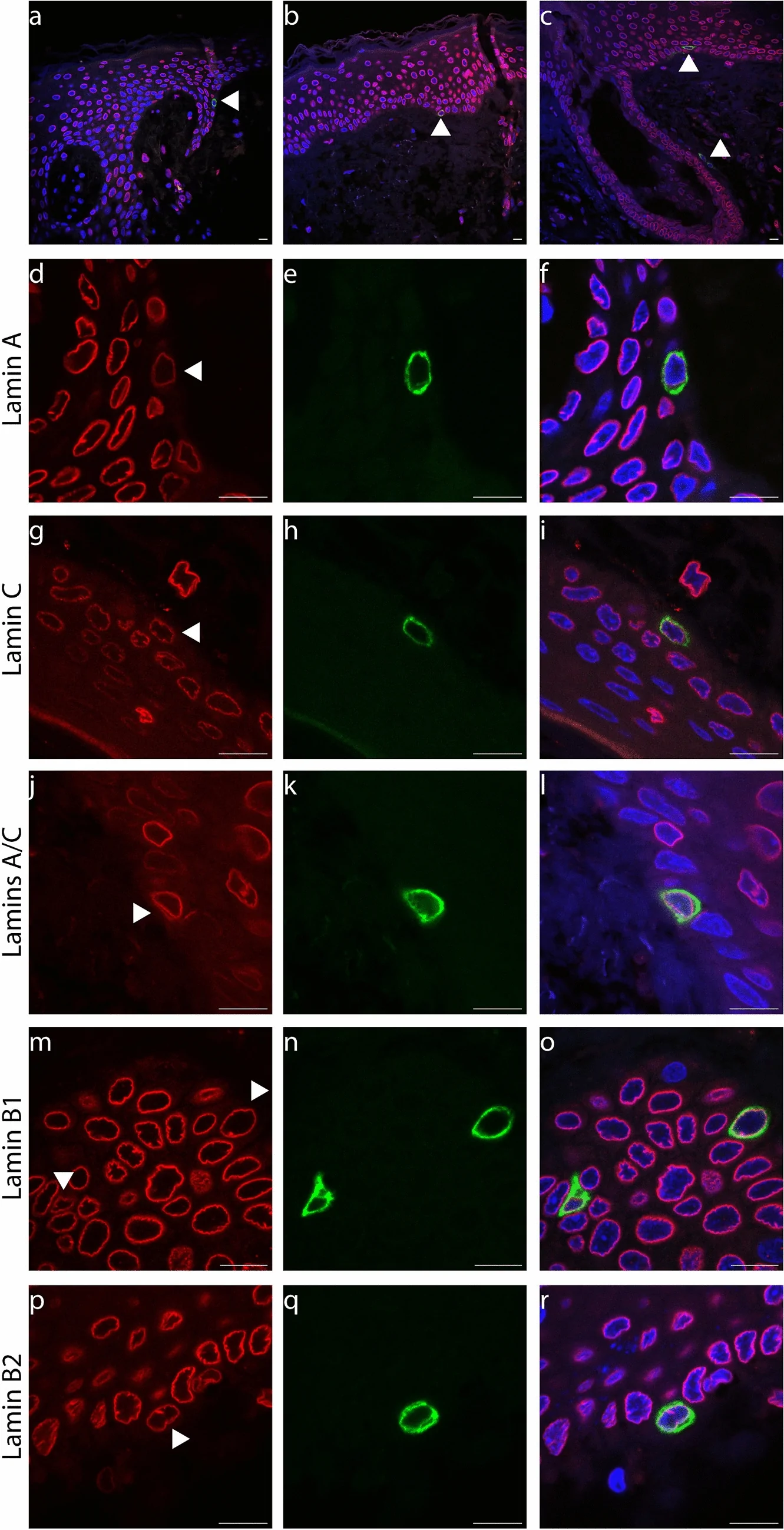
Double-label immunofluorescence of lamins (red) and cytokeratin 20 (green) in normal Merkel cells of human scalp skin, counterstained with DAPI (blue). **a**–**c** Low magnification confocal laser scanning microscopy (CLSM) images showing the location of normal Merkel cells. Arrowheads point to Merkel cells. **a** Lamin A, **b** and **c** lamin B1. **d**–**r** High-magnification CLSM images of immunostaining for individual lamins (left row), cytokeratin 20 (middle row), and merged images including blue DAPI staining of the nucleus (right row). **d**–**f** lamin A, **g**–**i** lamin C, **j**–**l** lamins A/C, **m**–**o** lamin B1, and** p–r** lamin B2. Note consistent nuclear lamina staining of Merkel cells (arrowheads) for all lamins studied. Further note basal layer enhancement of keratinocytes for lamin C. Scale bar indicates 10 µm

Because of the apparent flexibility that the MCC cells showed in their A-type lamin expression patterns, we wondered about the expression levels of these lamins in normal Merkel cells. Double-label immunofluorescence studies, combining cytokeratin 20 staining with the different lamin subtypes, revealed that both A- and B-type lamins were expressed in normal Merkel cells in human scalp skin (Fig. 6). In the 8–17 Merkel cells that could be studied in the available tissue samples, all demonstrated A-type (Fig. 6d–l) and B-type lamins (Fig. 6 m–r). In general, the nuclear A- and B-type lamin staining of Merkel cells was slightly variable but in most instances relatively strong, with the staining intensity being comparable to that of the surrounding keratinocytes.

## Discussion

In this study we demonstrate that MCC express variable, but mostly (very) low levels of A-type lamins, while the B-type lamins were overall highly expressed. No differences were detected between MCPyV-positive and -negative tumors. However, frequently a higher level of A-type lamins was detected in the lymph node metastases as compared with primary tumors, but when comparing the primary tumor and metastasis of the same patient, increased, decreased, or similar A-type lamin expression is observed. In a number of cases a higher staining intensity for the A-type lamins was observed at the margin of the tumor tissue as compared with the center. The implications of these findings are discussed in the following paragraphs.

### A-type lamin expression and MCC invasive and metastatic potential

The increased A-type lamin expression in a significant part of the metastases as compared with the primary tumors could point toward a need for a higher A-type lamin level during and/or after the process of migration of the tumor cells. Indeed, several studies have described that a high A-type lamin expression contributes to nuclear stability, which is necessary to withstand the mechanical stress that cells are exposed to during invasion of the surrounding stroma and for metastasis throughout the body (Broers et al. 2004; Lammerding et al. 2006; Mitchell et al. 2015; Wintner et al. 2020). In the current study, not all metastases demonstrated a higher IRS for A-type lamin expression as compared with the expression levels generally found for the primary tumors. In addition, in the seven paired cases of a primary tumor or local recurrence and their metastasis, upregulation, downregulation, and similar A-type lamin expression is seen. Strikingly, case MC17 demonstrates comparable A-type lamin expression levels in the primary tumor and in-transit metastasis, but higher lamin A expression in the liver metastasis. A recent study into lamin expression levels of serous ovarian carcinoma also revealed an increased lamin A expression in lymph node metastases, but not in peritoneal dissemination (Ouchi et al. 2023). The capacity of a cancer cell to metastasize could also be facilitated by a decrease in A-type lamin expression, instead of an increase, as this results in a more flexible nucleus that aids in migration through the extracellular matrix (Harada et al. 2014). However, loss of A-type lamin expression makes a cell more prone to cell death, due to nuclear ruptures and overall mechanical weakness of the cancer cell. 

MCC tissues demonstrate a high expression of apoptosis inhibitors, such as Bcl-2 and survivin (Knapp et al. 2012; Tucci et al. 2006), but necrotic areas are frequently observed (Andea et al. 2008; Mott et al. 2004), which could be (partly) explained by the generally low A-type lamin expression levels.

In conclusion, cancer cells dynamically regulate their lamin expression levels depending on the demand for either withstanding mechanical stress or facilitating invasion, which could also hold true for MCC as reflected in their variable A-type lamin expression patterns. Further understanding of the role of lamin expression changes upon metastasis may shed light on the role of nuclear lamins in the aggressive nature of other cancers as well. It is especially interesting to relate the current findings to those described for SCLC, which is also a neuroendocrine carcinoma and has a high metastatic potential. Its metastatic lesions in the skin highly resemble MCC based on histology, especially the small-cell type MCC (Nghiem and Jaimes 2007; Vilasi et al. 2024). Previous studies revealed a lack of or low lamin A expression levels in both SCLC cell lines and tissue sections of SCLC (Broers et al. 1993; Jia et al. 2019).

### Increased lamin expression at the tumor margin: Artificial effect or a real biological phenomenon?

Since a number of MCC cases showed a higher immunostaining reactivity for the lamins at the margins of the tumor tissue, the suspicion of an “edge effect” arose. This term is used for a false-positive reaction at the edge of a histological section, which is commonly observed in IHC (Bussolati and Leonardo 2008; McCampbell et al. 2019; Ward and Rehg 2014). This may result in an artificial differential staining around the edge of the tissue. The primary reasons for this effect include (1) a fixation-induced false-positive reaction at the edge of a histological section, where the tissue is unevenly fixed so that the edge is fixed more than the center; (2) drying of the antibody solution applied to the slide around the edges of the tissue; or (3) variability in the thickness of the tissue section. Notably, the effect of enhanced lamin immunostaining occurred also at the margin of tumor nodules, bordering at stromal tissues (Supplemental Fig. S2), making antibody solution drying (similar to that occurring at the tissue edge) unlikely. The variability in thickness was excluded by estimates of the thickness of different areas of the tumors using brightfield imaging with CLSM, which did not reveal a correlation between the intensity of lamin immunostaining and the thickness of the tissue within a given section (results not shown). Nevertheless, a slight compression of the soft cell-rich tumor tissue at its margins during sectioning resulting in enhanced immunostaining cannot be totally excluded. Repeated immunostaining of the same case and using different controls revealed a comparable phenomenon, which further minimizes the likelihood of the margin effect being due to antibody solution drying. In case the margin effect would be due to a fixation artifact, we expected to also observe an increased immunostaining intensity for other proteins, but we did not observe this for cytoplasmic constituents. In contrast, MSH2 and Ki-67, both nuclear proteins, did reveal such an enhanced immunostaining intensity at the margin of the tumor tissue. Possibly, nuclear proteins, including the nuclear lamins, are more prone to the margin effect as compared with cytoplasmic proteins. A previous study indeed suggested that nuclear antigens may be more sensitive to fixation and handling conditions than cytoplasmic antigens (Arber 2002). Our finding that the effect of increased lamin immunostainings occurred at the margin of tumor nodules, bordering at stromal tissues, at first glance might suggest a biological phenomenon. However, this effect was also occasionally found in normal control cells at the tissue margin such as normal lymph node tissue, which now rather might argue in favor of an artifact and against a biological phenomenon. In summary, no firm conclusions can yet be drawn regarding the nature of the enhanced lamin immunostaining levels at the tumor margin. It is of great importance to notice that these margin effects do not alter our general conclusions with respect to lamin expression levels in MCC.

### A-type lamins and treatment modalities for MCC

Taken together, the results of this study suggest a low A-type lamin expression in primary MCC. In addition, a differential lamin expression between primary tumor and metastases of MCC was found. Further research into the lamin expression levels in MCC is valuable not only for understanding cancer biology but also for treatment perspectives. A recent study correlated tumor sensitivity to paclitaxel with different A-type lamin expression levels (Smith et al. 2021). Cells with a low A-type lamin expression were found to be highly sensitive for paclitaxel treatment. In this respect it is interesting to note that a clinical trial into paclitaxel use in MCC was proposed in 2018, but could be not executed due to a lack of participating patients (Medicine 2018). These findings underscore the importance of additional research into the lamin expression levels in cancer, including MCC, and the relation between lamin expression and anti-cancer drug effectiveness.

### Does A-type lamin expression in normal Merkel cells indicate that they are not the cell of origin for MCC?

Our finding that normal Merkel cells express both A- and B-type lamins in apparently similar levels does not correspond with the A-type lamin expression levels in primary tumors, expressing low or no A-type lamin levels. These findings contribute to the discussion about the origin of Merkel-cell carcinoma (MCC) by challenging the traditional view of MCC originating from Merkel cells. As described above, the true cell of origin for MCC remains ambiguous (Harms et al. 2018). Merkel cells are located in the basal layer of the epidermis and hair follicle bulge. Although there are morphological and biological similarities between the Merkel cells and MCC, such as the presence of neuroendocrine markers and the positive immunostaining for cytokeratin 20, certain differences also exist. These include among others markers for early immune cell lineages (see below). Another argument against the Merkel cell as the cell of origin for MCC is that the malignancies are in general located in the dermis, sparing the epidermal structures. The finding that the normal Merkel cell expresses both lamins A and C adds another argument to the doubts surrounding its role in the development of MCC.

Apart from the normal Merkel cell, alternative progenitor candidates for MCC include epidermal and dermal stem cells, dermal fibroblasts, and pre-/pro-B-cells (Harms et al. 2018; Liu et al. 2016; Sauer et al. 2017). For example, Narisawa et al. (Narisawa et al. 2019) proposed the concept that both Merkel cells and MCC are derived from Merkel-cell progenitors residing within the hair follicle bulge, with a potential for neuroendocrine as well as epithelial differentiation. Earlier studies have also proposed a B-cell lineage as a possible origin for MCC, among others supported by the presence of markers such as PAX5 and TdT and expression of genes involved in immune cell function [5, 48]. An additional argument in support of this theory is the fact that this specific hematopoetic cell population was shown to be negative or only weakly positive for A-type lamins (Jansen et al. 1997; Saez et al. 2020).

In the case that mature Merkel cells turn out to be the cells of origin for MCC, as, for example, supported by the prediction of MCC to arise in the head and neck area, which has a relatively high density of Merkel cells [54], our findings may reflect a high degree of flexibility of these cells with respect to A-type lamin expression. Downregulation of A-type lamins during the early stages of Merkel-cell carcinogenesis should be taken into serious consideration, in light of our observation that their expression was found to be often low in primary tumors and high in metastatic MCCs. However, in paired cases both up- and downregulation in metastatic cases compared with the primary tumor has been observed. These findings indicate that these cells have the potency to adapt their lamin expression in response to changing conditions, as seen in the development of many other tumors (recent review Paganelli et al. 2025). Our finding adds another layer of data to consider when discussing the origin of MCC, which remains a complex and debated topic with no single hypothesis providing conclusive evidence until now.

## Supplementary Information

Below is the link to the electronic supplementary material.Supplementary file1 (DOCX 3673 KB)

## Data Availability

The major datasets generated and/or analyzed during the current study are presented and summarized in the text of this publication. Additional data are available from the corresponding author upon reasonable request.
