# Supplementary material for: Lamin subtype expression in Merkel-cell carcinoma: its relation to polyomavirus infection and tumor metastasis
Source: Histochem Cell Biol. 2026 Aug 27;164(1):79. doi: 10.1007/s00418-026-02526-1 (PMC13522004; doi:10.1007/s00418-026-02526-1)
Supplement: Supplementary file 1 [file 418_2026_2526_MOESM1_ESM.docx]

**Lamin Subtype Expression in Merkel Cell Carcinoma: Its Relation to Polyomavirus Infection and Tumor Metastasis**

Journal of Histochemistry and Cell Biology

Merel Stiekema^1,2*^, Monique Ummelen^1,2^, [Moritz Jesinghaus](https://pubmed.ncbi.nlm.nih.gov/?term=Jesinghaus+M&cauthor_id=38309431)^3^, [Corinna U. Keber](https://pubmed.ncbi.nlm.nih.gov/?term=Keber+CU&cauthor_id=35184226)^3^, Viktoria Wischmann^3^, Ingrid Moll^4^, Jack Cleutjens^5,6^, Marc A.M.J. van Zandvoort^1,2,6,7,8^, Jos L.V. Broers^1,2,6^, Frans C.S. Ramaekers^1,2^, Roland Moll^3^

^1^ Department of Genetics and Cell Biology, Maastricht University Medical Centre, Maastricht, the Netherlands;

^2^ GROW-Research Institute for Oncology and Reproduction, Maastricht University Medical Centre, Maastricht, the Netherlands;

^3^ Philipps-Universität Marburg, Institute of Pathology, Marburg, Germany;

^4^ Department of Dermatology and Venerology, University Hospital Hamburg-Eppendorf, Hamburg, Germany;

^5^ Department of Pathology, Maastricht University Medical Centre, Maastricht, the Netherlands;

^6^ CARIM-Cardiovascular Research Institute Maastricht, Maastricht University Medical Centre, Maastricht, the Netherlands;

^7^ Institute for Molecular Cardiovascular Research IMCAR, RWTH Aachen University, Aachen, Germany.

^8^ Institute for Mental Health and Neuroscience (MHeNS), Maastricht University Medical Centre, Maastricht, the Netherlands

*Correspondence:

Merel Stiekema

Department of Genetics and Cell Biology,

Maastricht University Medical Centre,

Universiteitssingel 50, 6229ER, Maastricht, The Netherlands

[m.stiekema@maastrichtuniversity.nl](mailto:m.stiekema@maastrichtuniversity.nl)

**SUPPLEMENTAL DATA**

**Supplemental Figure S1:** MCPyV-positive Merkel cell carcinoma cases display variable levels of positivity for MCPyV-LT immunostaining. Brightfield images of MCPyV-LT IHC in case (**a**) MC03, (**b**) MC06, (**c**) MC07, (**d**) MC13, (**e**) MC22, and (**f**) MC20A. The scores assigned are + for case MC06 and ++ for all other cases (Supplemental Table S1). Scale bar indicates 50 μm.

**Supplemental Figure S2:** Differential lamin expression patterns in the tumor margin and tissue edge as compared to the center of the tumor. **a**) Overview of case MC03, immunostained for lamin A. Black arrow points towards region with higher immunoreactivity in the tumor margin at the border to adjacent normal subcutaneous tissue, enlarged in **b-f**. Red arrow points towards region with higher immunoreactivity at the tissue edge (surface), enlarged in (**g-k**). **b-k**) Brightfield images of lamin A (**b, g**), lamin C (**c, h**), lamins A/C (**d, i**), lamin B1 (**e, j**), and lamin B2 (**f, k**) immunohistochemistry. Scale bar in **A** indicates 1 mm and 50 µm for **b-k**.

**Supplemental Figure S3:** Difference in A-type lamin expression in a primary tumor (case M11A), its lymph node metastasis (case MC11B) and its skin metastasis (case MC11C). Brightfield images of lamin A (**a, d, g**), lamin C (**b, e, h**), and lamins A/C (**c, f, i)**. **a-c**) Primary tumor, case MC11A. **d-f**) Lymph node metastasis, case MC11B. **g-i**) Skin metastasis, case MC11C. Scale bar indicates 50 μm.

**Supplemental Figure S4:** Difference in A-type lamin expression in a primary tumor (case M17A), its skin metastasis (case MC17B), and its liver metastasis (case MC17C). Brightfield images of lamin A (**a-c)** and lamins A/C (**d-f**). **a, d**) Primary tumor, case MC17A. **b, e**) Skin metastasis, case MC17B. **c, f**) Liver metastasis, case MC17C. Scale bar indicates 50 μm.

**Supplemental Table S1:** Clinical and immunohistochemical characteristics of the MCC cases investigated in this study. CK20: cytokeratin 20, LT: MCPyV large T antigen.

|  |  |  | **Immunohistochemical staining results^a^** | | | |  |
| --- | --- | --- | --- | --- | --- | --- | --- |
| **Case (Age/Sex)** | **Tumor location** | **Sample type** | **CK20** | **Chromogranin** | **Synaptophysin** | **LT** | **Small cell variant^b^** |
| MC01 (76/F) | Primary site (face) | Surgical resection | + | + | + | + | - |
| MC02A  (88/F) | Primary site (lower leg) | Surgical resection | + | + | + | - | - |
| MC02B  (88/F) | Lymph node | Needle biopsy | + (sparse) | n.d. | + | n.d. | - |
| MC03  (81/F) | Primary site (face) | Surgical resection | + | + | + | ++ | + |
| MC04  (71/M) | Lymph node | Surgical resection | + | + | + | ++ | - |
| MC05 (77/M) | Primary site (elbow) | Surgical resection | + | + | n.d. | ++ | - |
| MC06 (84/M) | Primary site (thigh) | Surgical resection | + | + | + | + | + |
| MC07  (73/M) | In-transit metastasis (thigh) | Surgical resection | + | + | - | ++ | - |
| MC08 (83/M) | Parotid gland metastasis | Surgical resection | + | + | + | ++ | - |
| MC09 (84/M) | Primary site  (upper arm) | Surgical resection | + | + | + | + | - |
| MC10 (76/M) | Primary site (thigh) | Surgical resection | + | + | + | + | - |
| MC11A (75/M) | Primary site (face) | Surgical resection | + (sparse) | + | + | - | + |
| MC11B  (77/M) | Lymph node | Surgical resection | + (sparse) | + (sparse) | + (sparse) | - | + |
| MC11C  (77/M) | Skin metastasis (temple) | Surgical resection | + (sparse) | + | + | - | + |
| MC12  (77/F) | Primary site (face) | Surgical resection | + | + | n.d. | ++ | - |
| MC13  (88/M) | Primary site (face) | Surgical resection | + | + | + | ++ | - |
| MC14A  (75/M) | Lymph node | Surgical resection | + | n.d. | + | ++ | - |
| MC14B  (75/M) | Lymph node | Surgical resection | + | n.d. | + | + | - |
| MC15 (62/M) | Primary site (forearm) | Surgical resection | + | + | n.d. | ++ | - |
| MC16  (83/M) | Lymph node | Surgical resection | + | + | + | - | - |
| MC17A  (78/M) | Primary site  (neck) | Surgical resection | + | + | + | - | + |
| MC17B  (78/M) | In-transit metastasis (neck) | Surgical resection | + | + | + | - | + |
| MC17C  (78/M) | Liver metastasis | Needle biopsy | + | + | + | - | + |
| MC18A  (82/F) | Local recurrence  (face) | Surgical resection | + | + | + | ++ | - |
| MC18B  (82/F) | Lymph node | Surgical resection | + | + | + | ++ | - |
| MC19  (53/F) | Lymph node | Needle biopsy | + | n.d. | + | ++ | - |
| MC20A (75/F) | Primary site (back of the knee) | Surgical resection | + | + (sparse) | n.d. | ++ | - |
| MC20B  (78/F) | Lymph node | Needle biopsy | + | + (sparse) | + | ++ | + |
| MC20C (75/F) | Skin metastasis (lower leg) | Surgical resection | + | + (sparse) | n.d. | + | - |
| MC21  (54/M) | Lymph node | Needle biopsy | + (sparse) | + | + | ++ | + |
| MC22  (78/F) | Lymph node | Needle biopsy | + | + | + | ++ | - |
| MC23  (70/M) | Lymph node | Needle biopsy | + | - | + | - | + |
| MC24  (85/F) | Primary site  (thigh) | Surgical resection | + | + | + | ++ | - |
| MC25  (72/M) | Primary site (thigh) | Surgical resection | + | + | + | + | - |
| MC26  (73/F) | Primary site (face) | Surgical resection | + | + | + | ++ | - |
| MC27 (69/M) | Primary site (forearm) | Surgical resection | + | + | + | +^c^ | - |
| MC28A (87/M) | Primary site (face) | Surgical resection | + | + | + | - | - |
| MC28B (87/M) | Lymph node | Surgical resection | + | n.d. | + | - | - |

^a^ Scoring immunohistochemical staining CK20/chromogranin A/synaptophysin: positive (+), negative (-) or not detected (n.d.). MCPyV-LT: - for <1% of nuclei stained, + for ≥1 and <5% of nuclei stained, ++ for ≥5% of nuclei stained.

^b^ Small cell variant: assessed by mean nuclear diameter, small cell variant (+) if the mean nuclear diameter is <7 μm, otherwise scored as -.

^c^ In addition to nuclear staining, cytoplasmic staining is observed.

**Supplemental Table 2:** Characteristics of primary antibodies used for brightfield immunohistochemical staining of FFPE MCC tissues.

| **Antibody** | **Antigen** | **Species, subclass** | **Dilution** | **Provider** | **Cat. No.** |
| --- | --- | --- | --- | --- | --- |
| 133A2 | Lamin A | Mouse monoclonal, IgG3 | 1:500 | Nordic-MUbio, Susteren, NL | MUB1101P |
| Jol2 | Lamins A/C | Mouse monoclonal, IgG1 | 1:20 | Abcam, Cambridge, UK | ab40567 |
| Lamin B1 | Lamin B1 | Rabbit polyclonal IgG | 1:2000 | Abcam, Cambridge, UK | ab16048 |
| 8F6-E8-F12 | Lamin B2 | Mouse monoclonal, IgG1,к | 1:200 | Biolegend, San Diego, USA | 871502 |
| Lamin C | Lamin C | Rabbit polyclonal IgG | 1:100 | Immuquest, Stokesley, UK | IQ177 |
| CM2B4 | MCPyV Large T antigen | Mouse recombinant monoclonal, IgG2b | 1:1000 | Abcam, Cambridge, UK | ab307450 |
| Ks20.8 | Cytokeratin 20 | Mouse monoclonal, IgG2a,к | 1:100 | Dako/Agilent, Waldbronn, DE | M7019 |
| DAK-SYNAP | Synaptophysin | Mouse monoclonal, IgG1,к | 1:600 | Dako/Agilent, Waldbronn, DE | M7315 |
| Chromogranin A | Chromogranin A | Rabbit polyclonal IgG | 1:2000 | Zytomed Systems, Berlin, DE | 503-1524 |
| MIB-1 | Ki-67 | Mouse monoclonal, IgG1,к | 1:100 | Dako/Agilent, Waldbronn, DE | M7240 |
| FE11 | MSH2 | Mouse monoclonal, IgG1,к | 1:25 | Zytomed Systems, Berlin, DE | MSK031-05 |

**Supplemental Table 3:** Characteristics of secondary antibodies used for brightfield immunohistochemical staining of FFPE MCC tissues.

| **Antibody** | **Dilution** | **Provider** | **Cat. No.** |
| --- | --- | --- | --- |
| Biotinylated horse anti-mouse IgG | 1:200 | Vector Laboratories, Burlingham, USA | BA-2001 |
| Biotinylated goat anti-rabbit IgG | 1:400 | Vector Laboratories, Burlingham, USA | BA-1000 |
| Bond Polymer Refine Detection Kit^a^ | - | Leica Biosystems, Wetzlar, DE | DS9800 |

^a^Used for detection of cytokeratin 20, synapthophysin, and chromogranin A with the Leica BOND-MAX automated immunostainer.

**Supplemental Table S4:** Characteristics of primary antibodies used for fluorescence immunohistochemical staining of FFPE normal human skin tissues.

| **Antibody** | **Antigen** | **Species, subclass** | **Dilution** | **Provider** | **Cat. No.** |
| --- | --- | --- | --- | --- | --- |
| 133A2 | Lamin A | Mouse monoclonal, IgG3 | 1:500 | Nordic-MUbio, Susteren, NL | MUB1101P |
| Jol2 | Lamins A/C | Mouse monoclonal, IgG1 | 1:20 | Abcam, Cambridge, UK | ab40567 |
| Lamin B1 | Lamin B1 | Rabbit polyclonal IgG | 1:4000 | Abcam, Cambridge, UK | ab16048 |
| 8F6-E8-F12 | Lamin B2 | Mouse monoclonal, IgG1,к | 1:200 | Biolegend, San Diego, USA | 871502 |
| Lamin C | Lamin C | Rabbit polyclonal IgG | 1:100 | Immuquest, Stokesley, UK | IQ177 |
| Ks20.8 | Cytokeratin 20 | Mouse monoclonal, IgG2a,к | 1:100 | Dako/Agilent, Waldbronn, DE | M7019 |

**Supplemental Table S5:** Characteristics of secondary antibodies used for fluorescent immunohistochemical staining of FFPE normal human skin tissues.

| **Antibody** | **Dilution** | **Provider** | **Cat. No.** |
| --- | --- | --- | --- |
| Goat anti Mouse IgG2a FITC | 1:50 | Southern Biotech | 1080-02 |
| Goat anti Mouse IgG3 Texas Red | 1:50 | Southern Biotech | 1100-07 |
| Goat anti Rabbit Ig Texas Red | 1:50 | Southern Biotech | 4010-07 |
| Goat anti Mouse IgG1 Texas Red | 1:50 | Southern Biotech | 1070-07 |

**Supplemental Table S6:** Semi-quantitative analysis of A- and B-type lamins immunohistochemical staining in MCC. For each case and lamin type the percentage of positive cells (P) and the staining intensity score (I) is displayed. The immunoreactive score (IRS) is obtained by multiplying P and I. The order of the cases has been rearranged to combine primary tumor samples and different types of metastases.

| **Carcinoma** | **Lamin A** | | | | | | | |  | |  | **Lamin C** | |  | |  | **Lamins A/C** | |  |  | **Lamin B1** | |  | |  | **Lamin B2** | | |  |  |
| --- | --- | --- | --- | --- | --- | --- | --- | --- | --- | --- | --- | --- | --- | --- | --- | --- | --- | --- | --- | --- | --- | --- | --- | --- | --- | --- | --- | --- | --- | --- |
| **Score** | P | | | | I | | | | | IRS | | P | I | | IRS | | P | I | IRS | | P | I | | IRS | | P | I | IRS | | |
| ***Primary tumor*** | | |  | |  | | | | |  | |  |  | |  | |  |  |  | |  |  | |  | |  |  |  | | |
| **MC01^a^** | 60% | | | | + | | | | | 3*1=1 | | 15% | + | | 2*1=2 | | 60% | + | 3*1=3 | | 100% | +++ | | 4*3=12 | | 100% | +++ | 4*3=12 | | |
| **MC02A** | 20% | | | | + | | | | | 2*1=2 | | 5% | + | | 1*1=1 | | 5% | + | 1*1=1 | | 100% | +++ | | 4*3=12 | | 100% | +++ | 4*3=12 | | |
| **MC03^a^** | 15% | | | | + | | | | | 2*1=2 | | 20% | + | | 2*1=2 | | 20% | + | 2*1=2 | | 100% | +++ | | 4*3=12 | | 100% | +++ | 4*3=12 | | |
| **MC05^a^** | 30% | | | | + | | | | | 2*1=2 | | 20% | + | | 2*1=2 | | 40% | + | 2*1=2 | | 100% | +++ | | 4*3=12 | | 100% | +++ | 4*3=12 | | |
| **MC06^a^** | 5% | | | | + | | | | | 1*1=1 | | 5% | + | | 1*1=1 | | 15% | + | 2*1=2 | | 100% | +++ | | 4*3=12 | | 100% | +++ | 4*3=12 | | |
| **MC09^a^** | 30% | | | | + | | | | | 2*1=2 | | 5% | + | | 1*1=1 | | 30% | + | 2*1=2 | | 100% | +++ | | 4*3=12 | | 100% | +++ | 4*3=12 | | |
| **MC10^a^** | 40% | | | | ++ | | | | | 2*2=4 | | 30% | + | | 2*1=2 | | 70% | ++ | 3*2=6 | | 100% | +++ | | 4*3=12 | | 100% | +++ | 4*3=12 | | |
| **MC11A** | 90% | | | | ++ | | | | | 4*2=8 | | 100% | ++ | | 4*2=8 | | 70% | ++ | 3*2=6 | | 100% | +++ | | 4*3=12 | | 100% | +++ | 4*3=12 | | |
| **MC12^a^** | 5% | | | | + | | | | | 1*1=1 | | 5% | + | | 1*1=1 | | 5% | + | 1*1=1 | | 100% | +++ | | 4*3=12 | | 100% | +++ | 4*3=12 | | |
| **MC13^a^** | 5% | | | | + | | | | | 1*1=1 | | 20% | + | | 2*1=2 | | 5% | + | 1*1=1 | | 100% | +++ | | 4*3=12 | | 100% | +++ | 4*3=12 | | |
| **MC15^a^** | 30% | | | | + | | | | | 2*1=2 | | 70% | + | | 3*1=3 | | 60% | + | 3*1=3 | | 100% | +++ | | 4*3=12 | | 100% | +++ | 4*3=12 | | |
| **MC17A** | 30% | | | | + | | | | | 2*1=2 | | 15% | + | | 2*1=2 | | 5% | + | 1*1=1 | | 100% | +++ | | 4*3=12 | | 100% | ++ | 4*2=8 | | |
| **MC20A^a^** | 60% | | | | + | | | | | 3*1=3 | | 90% | + | | 4*1=4 | | 60% | + | 3*1=3 | | 100% | +++ | | 4*3=12 | | 100% | +++ | 4*3=12 | | |
| **MC24^a^** | 40% | | | | + | | | | | 2*1=2 | | 0% | - | | 0*0=0 | | 0% | - | 0*0=0 | | 100% | +++ | | 4*3=12 | | 100% | +++ | 4*3=12 | | |
| **MC25^a^** | 60% | | | | + | | | | | 3*1=3 | | 5% | + | | 1*1=1 | | 60% | + | 3*1=3 | | 95% | +++ | | 4*3=12 | | 100% | ++ | 4*2=8 | | |
| **MC26^a^** | 40% | | | | + | | | | | 2*1=2 | | 2% | + | | 1*1=1 | | 15% | + | 2*1=2 | | 100% | ++ | | 4*2=8 | | 100% | ++ | 4*2=8 | | |
| **MC27^a^** | 75% | | | | + | | | | | 3*1=3 | | 0% | - | | 0*0=0 | | 40% | + | 2*1=2 | | 100% | ++ | | 4*2=8 | | 100% | ++ | 4*2=8 | | |
| **MC28A** | 90% | | | | ++ | | | | | 4*2=8 | | 45% | + | | 2*1=2 | | 60% | + | 3*1=3 | | 100% | +++ | | 4*3=12 | | 100% | +++ | 4*3=12 | | |
| ***Local recurrence*** | |  | | |  | | | | |  | |  |  | |  | |  |  |  | |  |  | |  | |  |  |  | | |
| **MC18A^a^** | 0% | | | | - | | | | | 0*0=0 | | 30% | + | | 2*1=2 | | 0% | - | 0*0=0 | | 100% | +++ | | 4*3=12 | | 100% | ++ | 4*2=8 | | |
| ***Lymph node metastasis*** | | | | | |  |  | | |  | |  |  | |  | |  |  |  | |  |  | |  | |  |  |  | | |
| **MC02B** | 40% | | | | + | | | | | 2*1=2 | | 0% | - | | 0*0=0 | | 20% | + | 2*1=2 | | 100% | +++ | | 4*3=12 | | 100% | +++ | 4*3=12 | | |
| **MC04^a^** | 90% | | | | +++ | | | | | 4*3=12 | | 30% | + | | 2*1=2 | | 85% | ++ | 4*2=8 | | 100% | +++ | | 4*3=12 | | 100% | +++ | 4*3=12 | | |
| **MC08^a,b^** | 40% | | | | + | | | | | 2*1=2 | | 0% | - | | 0*0=0 | | 40% | + | 2*1=2 | | 100% | +++ | | 4*3=12 | | 100% | +++ | 4*3=12 | | |
| **MC11B** | 5% | | | | + | | | | | 1*1=1 | | 70% | ++ | | 3*2=6 | | 30% | + | 2*1=2 | | 90% | +++ | | 4*3=12 | | 85% | +++ | 4*3=12 | | |
| **MC14A^a^** | 0% | | | | - | | | | | 0*0=0 | | 0% | - | | 0*0=0 | | 0% | - | 0*0=0 | | 100% | +++ | | 4*3=12 | | 100% | +++ | 4*3=12 | | |
| **MC14B^a^** | 0% | | | | - | | | | | 0*0=0 | | 0% | - | | 0*0=0 | | 0% | - | 0*0=0 | | 100% | +++ | | 4*3=12 | | 100% | +++ | 4*3=12 | | |
| **MC16** | 5% | | | | + | | | | | 1*1=1 | | 30% | + | | 2*1=2 | | 20% | + | 2*1=2 | | 100% | +++ | | 4*3=12 | | 100% | +++ | 4*3=12 | | |
| **MC18B^a^** | 0% | | | | - | | | | | 0*0=0 | | 60% | + | | 3*1=3 | | 30% | + | 2*1=2 | | 100% | +++ | | 4*3=12 | | 100% | +++ | 4*3=12 | | |
| **MC19^a^** | 60% | | | | + | | | | | 3*1=3 | | 90% | ++ | | 4*2=8 | | 85% | + | 4*1=4 | | 100% | +++ | | 4*3=12 | | 100% | +++ | 4*3=12 | | |
| **MC20B^a^** | 90% | | | | ++ | | | | | 4*2=8 | | 90% | ++ | | 4*2=8 | | 30% | + | 2*1=2 | | 100% | +++ | | 4*3=12 | | 100% | +++ | 4*3=12 | | |
| **MC21^a^** | 30% | | | | ++ | | | | | 2*2=4 | | 75% | ++ | | 3*2=6 | | 20% | + | 2*1=2 | | 100% | +++ | | 4*3=12 | | 100% | ++ | 4*2=8 | | |
| **MC22^a^** | 100% | | | | +++ | | | | | 4*3=12 | | 100% | ++ | | 4*2=8 | | 100% | ++ | 4*2=8 | | 100% | +++ | | 4*3=12 | | 100% | +++ | 4*3=12 | | |
| **MC23** | 85% | | | | ++ | | | | | 4*2=8 | | 100% | +++ | | 4*3=12 | | 60% | + | 3*1=3 | | 100% | +++ | | 4*3=12 | | 100% | +++ | 4*3=12 | | |
| **MC28B** | 70% | | | | + | | | | | 3*1=3 | | 30% | + | | 2*1=2 | | 45% | + | 2*1=2 | | 100% | +++ | | 4*3=12 | | 100% | +++ | 4*3=12 | | |
| ***Liver metastasis*** | | | | | |  | |  | |  | |  |  | |  | |  |  |  | |  |  | |  | |  |  |  | | |
| **MC17C** | 90% | | | | +++ | | | | | 4*3=12 | | 100% | + | | 4*1=4 | | 90% | ++ | 4*2=8 | | 100% | +++ | | 4*3=12 | | 100% | +++ | 4*3=12 | | |
| ***Skin metastasis*** | | | |  | | |  | | |  | |  |  | |  | |  |  |  | |  |  | |  | |  |  |  | | |
| **MC07^a,c^** | 20% | | | | + | | | | | 2*1=2 | | 0% | - | | 0*0=0 | | 15% | + | 2*1=2 | | 100% | +++ | | 4*3=12 | | 100% | +++ | 4*3=12 | | |
| **MC11C** | 60% | | | | + | | | | | 3*1=3 | | 60% | + | | 3*1=3 | | 30% | + | 2*1=2 | | 100% | +++ | | 4*3=12 | | 100% | +++ | 4*3=12 | | |
| **MC17B**^c^ | 40% | | | | + | | | | | 2*1=2 | | 60% | + | | 3*1=3 | | 30% | + | 2*1=2 | | 100% | +++ | | 4*3=12 | | 100% | +++ | 4*3=12 | | |
| **MC20C^a^** | 60% | | | | + | | | | | 3*1=3 | | 20% | + | | 2*1=2 | | 30% | + | 2*1=2 | | 100% | +++ | | 4*3=12 | | 100% | +++ | 4*3=12 | | |

*^a^ MCPyV-LT positive case*

*^b^Intraparotideal metastasis*

*^c^ In-transit metastasis*

**Supplemental Table S7:** Overview of immunoreactive scores (IRS) for A- and B-type lamin immunohistochemical staining of paired cases. The IRS is determined by multiplying the score for percentage of positive cells (0-4 points) with the staining intensity score (0-3 points); score is represented as: total (percentage of positive cells*intensity score).

| **Carcinoma** | **Tumor type** | **Lamin A** | **Lamin C** | **Lamins A/C** | **Lamin B1** | **Lamin B2** |
| --- | --- | --- | --- | --- | --- | --- |
| **MC02A** | Primary tumor | **2** (2*1) | **1** (1*1) | **1** (1*1) | **12** (4*3) | **12** (4*3) |
| **MC02B** | Lymph node metasasis | **2** (2*1) | **0** (0*0) | **2** (2*1) | **12** (4*3) | **12** (4*3) |
| **MC14A**^a^ | Lymph node metasasis | **0** (0*0) | **0** (0*0) | **0** (0*0) | **12** (4*3) | **12** (4*3) |
| **MC14B**^a^ | Lymph node metasasis | **0** (0*0) | **0** (0*0) | **0** (0*0) | **12** (4*3) | **12** (4*3) |
| **MC11A** | Primary tumor | **8** (4*2) | **8** (4*2) | **6** (3*2) | **12** (4*3) | **12** (4*3) |
| **MC11B** | Lymph node metastasis | **1** (1*1) | **6** (3*2) | **2** (2*1) | **12** (4*3) | **12** (4*3) |
| **MC11C** | Skin metastasis | **3** (3*1) | **3** (3*1) | **2** (2*1) | **12** (4*3) | **12** (4*3) |
| **MC17A** | Primary tumor | **2** (2*1) | **2** (2*1) | **1** (1*1) | **12** (4*3) | **8** (4*2) |
| **MC17B** | Skin metastasis | **2** (2*1) | **2** (2*1) | **2** (2*1) | **12** (4*3) | **12** (4*3) |
| **MC17C** | Liver metastasis | **12** (4*3) | **4** (4*1) | **8** (4*2) | **12** (4*3) | **12** (4*3) |
| **MC18A**^a^ | Local recurrence | **0** (0*0) | **2** (2*1) | **0** (0*0) | **12** (4*3) | **12** (4*3) |
| **MC18B**^a^ | Lymph node metasasis | **0** (0*0) | **3** (3*1) | **2** (2*1) | **12** (4*3) | **12** (4*3) |
| **MC20A**^a^ | Primary tumor | **3** (3*1) | **4** (4*1) | **3** (3*1) | **12** (4*3) | **12** (4*3) |
| **MC20B**^a^ | Lymph node metastasis | **8** (4*2) | **8** (4*2) | **2** (2*1) | **12** (4*3) | **12** (4*3) |
| **MC20C**^a^ | Skin metastasis | **3** (3*1) | **2** (2*1) | **2** (2*1) | **12** (4*3) | **12** (4*3) |
| **MC28A** | Primary tumor | **8** (4*2) | **2** (2*1) | **3** (3*1) | **12** (4*3) | **12** (4*3) |
| **MC28B** | Lymph node metasasis | **3** (3*1) | **2** (2*1) | **2** (2*1) | **12** (4*3) | **12** (4*3) |

*^a^ MCPyV-LT positive cases*
